# Development and Validation of a Stability-Indicating LC-Method for the Simultaneous Estimation of Levodropropizine, Chloropheniramine, Methylparaben, Propylparaben, and Levodropropizine Impurities

**DOI:** 10.3797/scipharm.1210-18

**Published:** 2012-11-17

**Authors:** Palakurthi Ashok Kumar, Thummala Veera Raghava Raju, Dongala Thirupathi, Ravindra Kumar, Jaya Shree

**Affiliations:** 1 Analytical Research and Development, Integrated Product Development, Dr. Reddy’s Laboratories Ltd., Bachupally, Hyderabad-500 072, India.; 2 Centre for Chemical Science and Technology, J. N. T. University, Kukatpally, Hyderabad, A.P., India.

**Keywords:** L-Dropropizine, Chloropheniramine, HPLC, ICH Guidelines, Development, Validation

## Abstract

A simple, fast, and efficient RP-HPLC method has been developed and validated for the simultaneous estimation of Levodropropizine, Chloropheniramine, Methylparaben, Propylparaben, and the quantification of Levodropropizine impurities in the Reswas syrup dosage form. A gradient elution method was used for the separation of all the actives and Levodropropizine impurities by using the X-Bridge C18, 150 mm × 4.6 mm, 3.5 μm column with a flow rate of 1.0 mL/min and detector wavelength at 223 nm. The mobile phase consisted of a potassium dihydrogen orthophosphate buffer and acetonitrile. All the peaks were symmetrical and well-resolved (resolution was greater than 2.5 for any pair of components) with a shorter run time. The limit of detection for Levodropropizine and its Impurity B was 0.07 μg/ml & 0.05 μg/ml, whereas the limit of quantification was 0.19 μg/ml & 0.15 μg/ml respectively. The method was validated in terms of precision, accuracy, linearity, robustness, and specificity. Degradation products resulting from the stress studies were well-resolved and did not interfere with the detection of Levodropropizine, Chloropheniramine, Methylparaben, Propylparaben, and Levodropropizine Impurity B, thus the test method is stability-indicating. Validation of the method was carried out as per International Conference on Harmonization (ICH) guidelines.

## Introduction

**Fig. 1:**
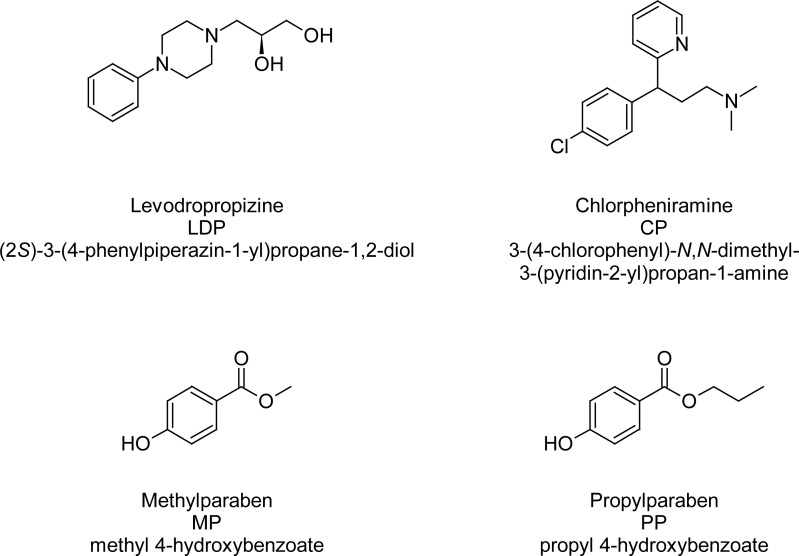
Structures and IUPAC names of LDP, CP, MP, PP

Levodropropizine (LDP), a phenylpiperazinopropane derivative, is a non-opioid antitussive agent. Chemically, levodropropizine is (2*S*)-3-(4-phenylpiperazin-1-yl)propane-1,2-diol and is the ‘levo’-isomer of dropropizine. Levodropropizine has been proven to be an effective antitussive against non-productive cough associated with different lung pathologies [1]. Chloropheniramine (CP) is a first-generation antihistamine belonging to the class of alkylamines. It is used in the prevention of symptoms of allergic conditions such as rhinitis and utricaria. Its sedative effects are relatively weak compared to other first-generation antihistamines. It is used not only for the treatment of cough, but also for other related allergy symptoms such as sneezing, itchy and/or watery eyes, itchy nose or throat, and runny nose caused by hay fever (allergic rhinitis), or other respiratory allergies. Methylparaben (MP) and Propylparaben (PP) are the preservatives used to prevent decomposition by microbial growth or by undesirable chemical changes. Preservatives can desirably be incorporated into the composition to protect against the growth of potentially harmful microorganisms. Microorganisms tend to grow in an aqueous phase, so to prevent their growth, a preservative has to be added to the pharmaceutical composition. The chemical structures of all the actives are shown in Fig. 1.

The objective of this work is to develop and validate a simple, precise, and accurate stability-indicating HPLC method for the estimation of LDP, CP, MP, PP, and Impurity B of LDP with a shorter run time. Literature surveys revealed that there is no HPLC method for the simultaneous determination of LDP, CP, MP, PP, and Impurity B of LDP. Several analytical methods such as liquid chromatography with a UV spectrophotometer [2–7], have been reported for the determination of LDP and CP individually. According to the literature [8], Levodropropizine has three impurities: Impurity A (Dextropropizine), Impurity B (1-Phenylpiperazine), Impurity C (Glycidol). Impurity A & C were estimated by the normal-phase-HPLC & GC respectively. Impurity B and any other unknown impurities were estimated by the RP-HPLC method.

## Experimental

### Chemicals, reagents, and samples

LDP Impurity B was obtained from Spectro Chem. India PVT Ltd. LC grade potassium dihydrogen orthophosphate and 1-Hexanesulphonic acid sodium salt, methanol, and acetonitrile were purchased from Merck India Limited. Pure water was prepared by using a Millipore Milli-Q Plus Water Purification System (Bedford, MA, USA).

### Equipments

The LC system was a Waters model 2996 equipped with a PDA Detector (Waters Corporation, Milford, USA). The output signal was monitored and processed using Empower software (Waters Corporation, Milford, USA) on a Pentium computer (Digital Equipment Co.).

### Chromatographic conditions

The chromatographic column X-Bridge C18 column (150 × 4.6) mm with 3.5μm particles was used. Mobile phase-A consisted of a mixture of 0.025M aqueous potassium dihydrogen orthophosphate buffer and 0.005M 1-Hexanesulphonic acid sodium salt buffer. Mobile phase-B consisted of a mixture of water and acetonitrile (10:90, v/v). The mobile phase was filtered through a 0.45 μm nylon membrane filter. The flow rate of the mobile phase was 1.0mL/min with a column temperature of 40°C and the detection wavelength at 223nm. The injection volume was 10μL. A water and methanol (80:20, v/v) mixture was used as a diluent during the preparation of the standard and sample. The LC gradient programme is as follows:

**Table:** 

**Time**	**%A**	**%B**	**Time**	**%A**	**%B**
0 min	80	20	12 min	90	10
5 min	45	55	13 min	80	20
10 min	90	10	16 min	80	20

### Preparation of Standard

#### CP and PP stock preparation

0.4 mg/mL of CP and 0.2 mg/mL of PP solution were prepared in methanol.

#### LDP and MP stock preparation

6.0 mg/mL of LDP and 2.0mg/mL of MP solution were prepared in methanol.

#### Standard preparation (For assay)

10mL of each of the above two stock solutions were transferred into a 100mL volumetric flask and diluted to volume with water and mixed well.

#### Standard preparation (For impurities)

0.3 mg/mL of the LDP standard solution was prepared in diluent. Then 1.0mL of this solution was further transferred to a 100mL volumetric flask and diluted to volume with diluent and mixed well.

### Sample Preparation

About 10mL of the syrup was weighed and transferred into a 100 mL volumetric flask, to which was added about 70mL of diluent. It was then sonicated for 5min and diluted to volume with diluent and mixed well.

## Results and Discussion

### Optimization of chromatographic conditions

The main difficulty in this study was to get symmetrical peaks for all the actives and better separation between LDP Impurity B and LDP in a single method without any interference. The presence of the amine group functionality in LDP and CP led to peak tailing. Initially as part of the method development, different stationary phases like C8, C18 were tried by using the gradient elution method with a phosphate buffer and found that the peak shapes were not symmetrical (more tailing observed). Different ionic strengths (ranging from 0.025M to 0.1M) were employed with phosphate, acetate, perchlorate, and formic acid buffers to reduce the peak tailings, but the ionic strength did not play any role in peak tailing. From the above experiments, it was observed that getting a symmetrical peak for the LDP buffer played a role and for CP, MP, and PP the organic phase played a role. We tried different gradient programmes by increasing the organic phase, where LDP and LDP Impurity B were merging.

**Fig. 2:**
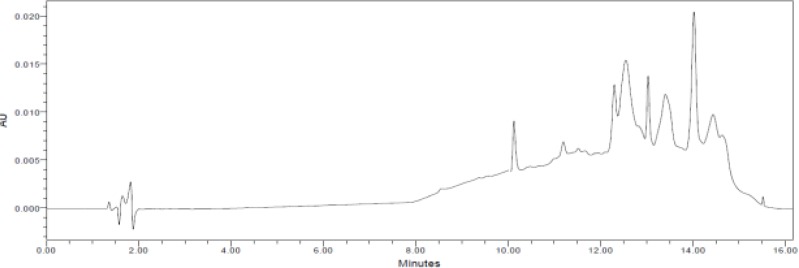
A typical chromatogram of the blank

**Fig. 3:**
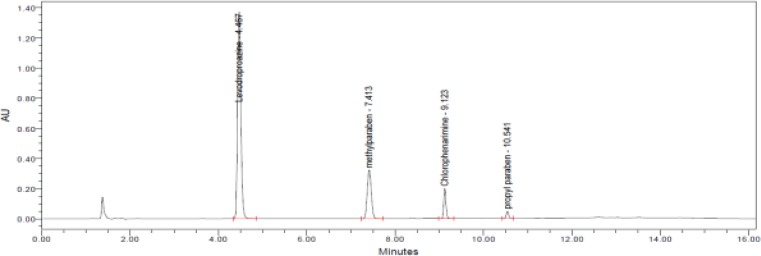
A typical chromatogram of the assay standard

**Fig. 4:**
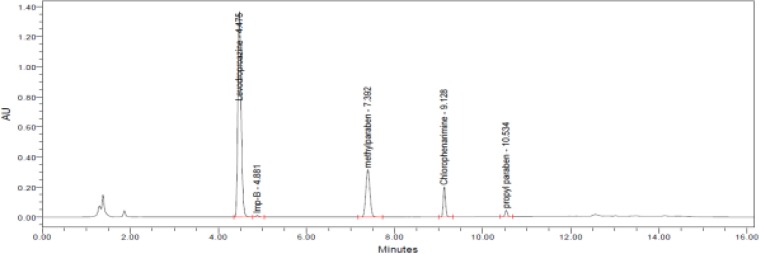
A typical chromatogram of the Impurity-spiked sample

**Fig. 5:**
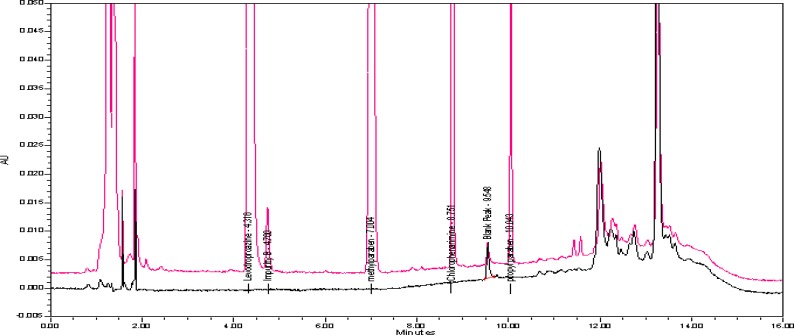
A typical overlaid zoomed chromatogram of the blank and spiked sample

We further tested different chromatographic parameters like column temperature (40°C to 60°C), column particle size (5μm & 3.5μm), pH (3.5 to 7.5) of buffer, and ion pair reagent incorporation in the mobile phase buffer to improve peak shapes and resolution. Finally, the chromatographic separation was achieved by a reverse phase X-Bridge C18 150 x 4.6 mm, 3.5μm particle size column operated at 40°C with gradient elution at 1.0 mLmin^−1^ using mobile phase A as a mixture of 0.025M aqueous potassium dihydrogen orthophosphate and 0.005M 1-Hexanesulphonic acid sodium salt in 1000ml water. Mobile phase B consisted of a mixture of water and acetonitrile (10:90, v/v). The detection wavelength was at 223nm and the injection volume was 10μL. The LC gradient program was set as: time (min)/% mobile phase B: 0.01/20, 5/55, 10/10, 12/10, 13/20, and 16/20. All the peaks (including active, blank, and the Impurity) were well-separated with a resolution greater than 2.5. No chromatographic interference was observed due to the blank (diluent) and other excipients (placebo) at the retention time of the active peaks and their impurities. Typical chromatograms are shown in Fig. 2–5.

### Establishment of Relative Response Factor for LDP Impurity B

We prepared and injected a series of solutions consisting of LDP Impurity B and LDP in the range of 0.4% to 1.2% (0.4%, 0.6%, 0.8%. 1.0%, 1.2%). The load of 10 μl injection volume of the sample was injected into the liquid chromatograph and we then calculated the Relative Response Factor (RRF) of LDP Impurity B with respect to LDP from the calibration curve data. The RRF value of LDP Impurity B was found to be 1.26. The Relative Retention Time (RRT) of LDP Impurity B was found to be 1.1

## Method Validation

After satisfactory development of the method, it was subjected to method validation as per ICH guidelines [9]. The method was validated to demonstrate that it is suitable for its intended purpose by the standard procedure to evaluate adequate validation characteristics (system suitability, specificity, accuracy, precision, linearity, robustness, ruggedness, solution stability, LOD and LOQ, and stability-indicating capability).

Specificity, linearity, precision, accuracy, robustness, and ruggedness were done as a part of the method validation.

### System suitability

The system suitability parameters (Table 1) were evaluated by making the injection of the assay standard and the RS standard. The system was deemed to be suitable as the tailing factors for all the peaks were between 0.8 to 1.5 and the resolution between LDP and LDP Impurity B was >2.5.

### Specificity

In order to determine whether the developed analytical method was stability-indicating, Reswas syrup and the LDP active pharmaceutical ingredient (API) was stressed under various conditions to conduct forced degradation studies. LDP is very soluble in water. The specificity was examined by analyzing the solution of a placebo, which consisted of all the excipients as per ICH guidelines [9]. The degradation study conducted for Levodropropizine used stress conditions like UV light, sunlight, thermal stress, water hydrolysis, acid hydrolysis, base hydrolysis, oxidation and humidity. The acidic, basic, and oxidative stress condition studies were carried out by refluxing the syrup for 6hours with 5N HCl, 0.1NNaOH, and 3% hydrogen peroxide respectively. The thermal stress was carried out by heating the drug product to 105°C for 24hours and the photodegradation was performed by exposing the drug product to 1.2million lux hours and 200 watt hours/m^2^ in a photostability chamber. It is interesting to note that all the peaks due to degradation were well-resolved. The chromatograms of the stressed samples were evaluated for peak purity using Waters Empower Networking Software. For all forced degradation samples, the purity angle was found to be less than the threshold angle and there was no purity flag for the LDP and its impurities. This confirms the stability-indicating power of the developed method. The results of the forced degradation study are shown in Table 2.

### Precision

The precision of the test method was evaluated by analyzing six samples of Reswas syrup by spiking the LDP Impurity B at a target concentration level (i.e. 0.5%) with respect to the test concentration of LDP (0.6mg/mL). The % R.S.D of LDP, LDP Impurity B, MP, CP, and PP from six sample preparations was found to be below 2.0 which indicates the precision of the method for the quantification of LDP, LDP Impurity B, MP, CP, and PP. The results are shown in Table 4.

### Limit of detection and quantification

A series of different concentrations of LDP and LDP Impurity B solutions was prepared in diluent. The limit of detection and limit of quantification were established based on a signal-to-noise ratio. The LOQ concentrations for LDP & LDP Impurity B were found to be 0.032% & 0.025%. Concentrations at the LOD and LOQ levels are reported in Tab. 5.

### Linearity

**Fig. 6:**
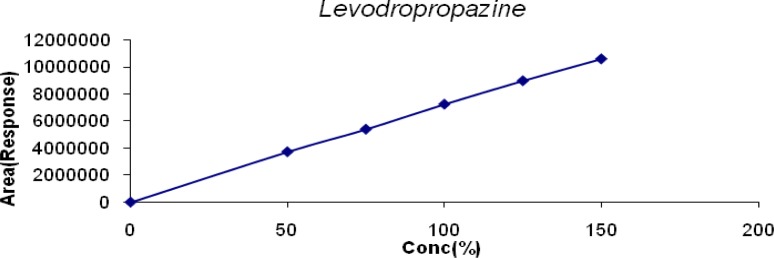
Linearity of Detector Response of LDP

**Fig. 10:**
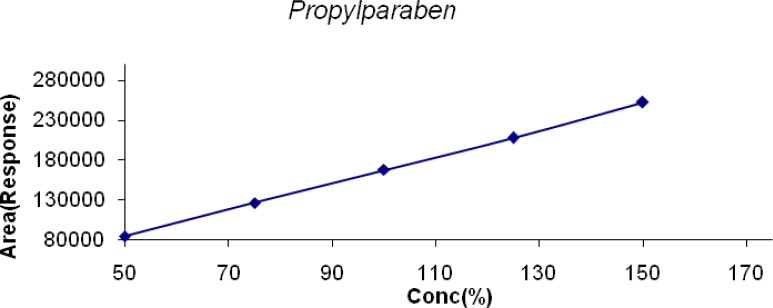
Linearity of Detector Response of PP

The linearity of LDP and LDP Impurity B was conducted from the LOQ level to 150% and for MP, CP, PP it was conducted from 50% to 150% of the target concentration (0.6mg/mL, 0.04mg/mL, 0.2mg/mL & 0.02mg/mL for LDP, CPM, and MP & PP respectively). The linearity graphs were plotted for concentration (%) versus detector response (area). The correlation coefficient for all the peaks was found to be 0.999. Linearity plots are shown in Fig. 6 to Fig. 10.

### Accuracy

The accuracy study for LDP and LDP Impurity B was conducted from the LOQ level to 150% and for MP, CP, PP it was conducted from 50% to 150% by spiking the APIs of LDP, CP, MP & PP and LDP Impurity B at different levels starting from the LOQ to 150% of the target concentration level (i.e. 0.6mg/mL, 0.04mg/mL, 0.2mg/mL, 0.02mg/mL & 0.003mg/mL, for LDP, CP, MP, PP & LDP Impurity B respectively). % recovery of LDP, CPM, MP, PP & LDP Impurity B was found to be between 95% to 105%. Results are tabulated in Table 6.

### Robustness

The robustness was investigated by varying the conditions with respect to a change in the flow rate, column temperature, and acetonitrile composition in mobile phase B. The study was conducted at the different flow rates of 0.8ml/min and 1.2ml/min instead of 1.0ml/min to study the effect of the change in flow rate. The column temperature was adjusted to 35°C and 45°C instead of the 40°C initial column oven temperature. The organic phase composition (acetonitrile) was studied from 90% to 110% in mobile phase B to study the effect of the organic phase composition variation in the mobile phase. The method was found to be robust with respect to flow rate, column temperature, and organic phase composition without any changes in system suitability parameters such as tailing factor and resolution (Table 7).

## Conclusion

A simple, selective, gradient mode high-performance liquid chromatographic method provides the selective quantification of LDP, LDP Impurity B, MP, CP, and PP without interference from the blank, placebo, and any other degradants, thereby affirming the stability-indicating nature of the method. The proposed method is highly selective, reproducible, specific, and rapid. This developed method can be applied successfully to the quality control of commercial and routine analysis. The information presented herein could be very useful for monitoring the quality of bulk samples as well as checking the quality during stability studies.

## Figures and Tables

**Fig. 7:**
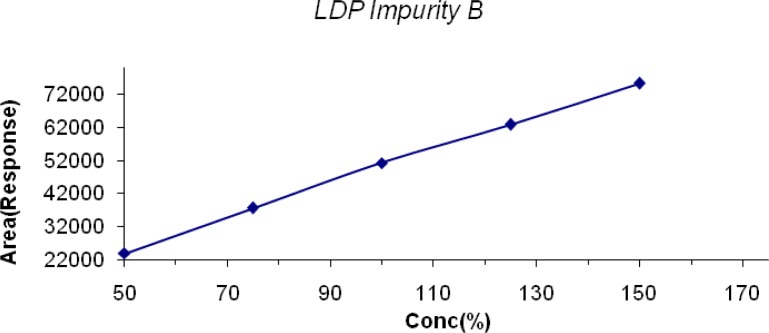
Linearity of Detector Response of LDP IMP B

**Fig. 8:**
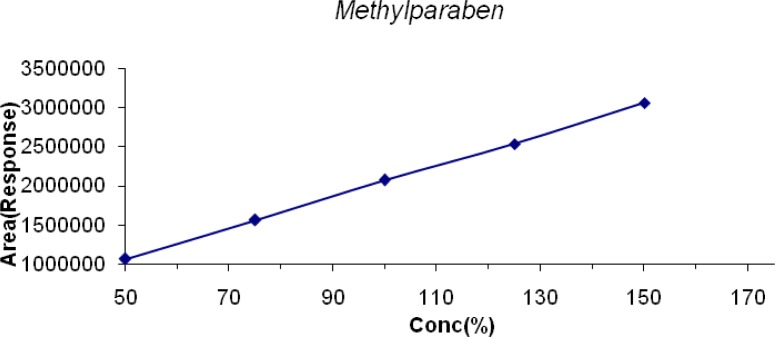
Linearity of Detector Response of MP

**Fig. 9:**
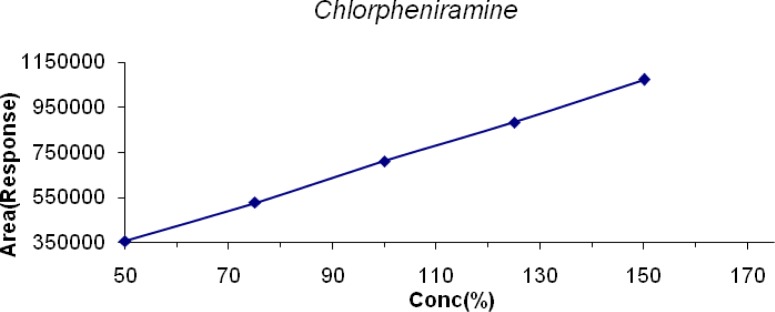
Linearity of Detector Response of CP

**Tab. 1.** The results table for System Suitability of the Assay standard

**Component**	**Tailing factor**	**USP plate count**	**% RSD**
Levodropropazine	1.3	16364	0.7
Methylparaben	1.0	28198	0.7
Chlorpheniramine	1.2	254055	0.8
Propylparaben	1.1	21567	0.8

**Tab. 2.** The results table for the specificity of Levodropropazine

**Sample No.**	**Stress Conditions**	**Purity angle**	**Purity threshold**	**Purity flag**
1	As such sample	1.135	3.215	NO
2	Acid stress	0.899	3.371	NO
3	Base stress	1.079	3.282	NO
4	Oxidation stress	0.718	1.250	NO
5	Sunlight stress	1.023	3.125	NO
6	UV light stress	1.158	3.098	NO
7	Thermal stress	0.963	3.264	NO
8	Humidity stress	1.564	3.598	NO
9	Water stress	1.132	3.201	NO

**Tab. 3.** The results table for the specificity of Levodropropazine Impurity B

**Sample No.**	**Stress Conditions**	**Purity angle**	**Purity threshold**	**Purity flag**
1	As such sample	0.566	0.792	NO
2	Acid hydrolysis	0.614	0.785	NO
3	Base hydrolysis	0.610	0.674	NO
4	Oxidation	0.645	0.831	NO
5	Sunlight stress	0.592	0.801	NO
6	UV light stress	0.621	0.798	NO
7	Thermal stress	0.658	0.804	NO
8	Humidity stress	0.603	0.765	NO
9	Water hydrolysis	0.575	0.691	NO

**Tab. 4.** The results table of the precision of the test method

**Component**	**Average**	**%RSD**
% Assay of Levodropropazine	102.3	1.0
% of LDP IMP B	0.49	2.6
% Assay of Methylparaben	100.5	1.1
% Assay of Chlorpheniramine	100.9	1.7
% Assay of Propylparaben	96.2	1.1

**Tab. 5.** LOD and LOQ of LDP and LDP IMP B

**Name of the component**	**Test Name**		**S/N Ratio**		**% at LOQ**

	**Conc. at LOD (μg/ml)**	**Conc. at LOQ (μg/ml)**	**LOD**	**LOQ**	
LDP	0.07	0.19	3.15	10.32	0.032
LDP IMP B	0.05	0.15	3.30	9.91	0.025

**Tab. 6.** The results table of the Accuracy of the test method

**Spike level**	**Levodropropazine**		**LDP IMP B**		**Methylparaben**		**Chlorpheniramine**		**Propylparaben**	

	**Mean**	**%RSD**	**Mean**	**%RSD**	**Mean**	**%RSD**	**Mean**	**%RSD**	**Mean**	**%RSD**
LOQ%	0.032	1.4	0.025	1.1	NA	NA	NA	NA	NA	NA
50%	102.3	0.1	0.234	0.9	100.9	0.2	102.4	0.4	99.2	0.4
75%	102.4	0.4	0.352	1.6	100.3	0.4	103.9	0.7	100.2	0.4
100%	101.2	0.4	0.540	1.6	99.2	0.4	104.5	0.3	99.2	0.3
125%	100.0	0.4	0.648	0.9	98.5	0.3	104.0	0.2	98.4	0.4
150%	98.4	0.2	0.734	1.5	99.2	0.1	105.2	0.2	99.9	0.2

**Tab. 7.** System suitability results from robustness

**Parameter**	**Flow rate (ml/min)**			**Column temperature (°C)**			**Organic phase (ACN) variation (±10%)**		

	**0.8**	**1.0**	**1.2**	**35**	**40**	**45**	**90**	**100**	**110**
Tailing factor for LDP peak	1.5	1.3	1.5	1.2	1.3	1.2	1.2	1.3	1.2
Resolution between LDP& LDP IMP B	3.4	3.4	3.6	2.8	3.4	2.8	2.8	3.4	2.8
Tailing factor for MP peak	1.0	1.0	1.0	1.1	1.0	1.1	1.1	1.0	1.1
Tailing factor for CP peak	1.2	1.2	1.2	1.1	1.2	1.1	1.1	1.2	1.1
Tailing factor for PP peak	1.0	1.0	1.0	1.0	1.0	1.1	1.0	1.0	1.0
